# A comparison of results of SRS-30 questionnaire in scoliosis patients treated surgically or conservatively

**DOI:** 10.1186/1748-7161-7-S1-P15

**Published:** 2012-01-27

**Authors:** H Yilmaz, T Kuru

**Affiliations:** 1 Canakkale Onsekiz Mart University Physical Medicine and Rehabilitation Department, Canakkale, Turkey; 2 Physical Therapy and Rehabilitation School, Istanbul, Turkey

## Background

The Scoliosis Research Society Questionnaire (SRS-30) is a specific instrument to measure health-related quality of life in patients with scoliosis who had undergone surgery or had not had. The objectives of this paper are evaluate pain, function, self-image, mental health and satisfaction with management of scoliosis patients and compare of surgical and conservative treatment.

## Materials and methods

The questionnaire was fulfilled by 80 individuals with scoliosis on the internet and we evaluated their answers. The mean age of the participants at the time they received the questionnaire was 18.6±4,2 years (min:12years, max:36years). 35 patients were treated with surgery and 45 were treated with conservative management (brace or/and exercise). 18 individuals were male and 62 individuals were female. Data analysis were performed using SPSS, version 12.

## Results

There were no differences between pain, function, self-image, mental health and satisfaction with management scores of patients who had surgery or not had (Table 1).

**Table 1:** 

Subgroups	Patients (n=80)	Mean ± SD	P value
**Pain**	Surgical treatment (n=35)	4.09±0.78	0.376
		
	Conservative treatment (n=45)	3.92±0.61	

**Function**	Surgical treatment (n=35)	3.83±0.87	0.433
		
	Conservative treatment (n=45)	3.99±0.71	

**Self-image**	Surgical treatment (n=35)	3.17±0.81	0.558
		
	Conservative treatment (n=45)	3.31±0.01	

**Mental health**	Surgical treatment (n=35)	3.08±0.98	0.791
		
	Conservative treatment (n=45)	3.15±0.83	

**Satisfaction with management**	Surgical treatment (n=35)	1.52±0.53	0.612
		
	Conservative treatment (n=45)	1.45±0.55	

## Conclusions

The data obtained in this study there were no significant differences in pain, function, self-image, mental health and satisfaction with management scores between the two groups although our study showed that surgery or conservative treatment does not implement successful patient satisfaction.

